# Routine antenatal molecular testing for α-thalassemia at a tertiary referral hospital in China: ten years of experience

**DOI:** 10.3389/fgene.2024.1416047

**Published:** 2024-06-04

**Authors:** Dongming Li, Lifang Liang, Dahua Meng, Sheng He

**Affiliations:** ^1^ Department of Laboratory Medicine, Maternal and Child Health Hospital of Guangxi Zhuang Autonomous Region, Nanning, China; ^2^ Guangxi Key Laboratory of Reproductive Health and Birth Defect Prevention, Maternal and Child Health Hospital of Guangxi Zhuang Autonomous Region, Nanning, China; ^3^ Department of Clinical Genetics, Maternal and Child Health Hospital of Guangxi Zhuang Autonomous Region, Nanning, China

**Keywords:** α-thalassemia, antenatal screening, hematological parameters, prenatal diagnosis, cost-saving

## Abstract

**Objective:**

This study aimed to evaluate the efficacy of α-thalassemia gene testing as a part of an antenatal intervention program over a 10-year period.

**Methods:**

All patients underwent α-thalassemia gene testing, which included the analysis of three types of deletions and mutations. Rare α-thalassemia gene testing was performed using Sanger sequencing, multiplex ligation-dependent probe amplification, and sequencing techniques. Prenatal diagnosis was performed in high-risk couples using chorionic villus sampling or amniocentesis.

**Results:**

From 2010 to 2019, among the 91,852 patients examined, α-thalassemia mutations were identified in 41.78% of patients. The most frequent α^0^ gene mutation was--^SEA^, followed by--^THAI^. Two rare α^0^-thalassemia gene mutations at --^32.8^ and --^230^, were also observed. A total of 2,235 high-risk couples were identified, of which 562 were affected, including three with the--^SEA^/--^THAI^ genotype and one with the--^SEA^/--^230^ genotype. Additionally, prenatal diagnosis revealed four cases of fetal anemia and/or mild edema, along with two cases of severe fetal edema. Chromosome and gene chip results were normal. Thalassemia gene testing showed an α^CS^α/α^CS^α genotype in four patients with anemia and/or mild edema, while two patients with severe fetal edema had one--^SEA^/α^CS^α genotype and one--^SEA^/--^GX^ genotype. Using the cut-off points of 74.6 fL and 24.4 pg as criteria for identifying α^0^-thalassemia carriers and HbH disease, the detection rate of missed diagnoses in high-risk couples is consistent with national guidelines for standards, potentially saving 10,217,700 ¥.

**Conclusion:**

Routine molecular testing for α-thalassemia in high-risk prenatal populations effectively prevented severe α-thalassemia births. Despite the high cost, the cutoff points proposed by this study suggest that implementing screening using a new parameter has the potential to reduce current expenses.

## 1 Introduction

α-Thalassemia is a common genetic disorder caused by a defect in the α-globin gene, resulting in hereditary hemolytic anemia. It is the primary cause of fetal hydrops in Southeast Asia, accounting for 33%–62% of cases (He et al., 2017a; Chainarong et al., 2021). Currently, long-term blood transfusion and hematopoietic stem cell transplantation are primary treatment options for severe thalassemia (Songdej et al., 2017; King and Higgs, 2018; Chan et al., 2021). Therefore, it is crucial to implement interventions that target high-risk populations to prevent the birth of children with severe α-thalassemia. Prenatal screening programs are established in countries with a high prevalence of thalassemia (Sorour et al., 2007; Bhukhanvala et al., 2013; Jiang et al., 2021). These programs typically involve initial tests, such as measuring red blood cell indices and quantifying hemoglobin(Hb)A2 levels. Although quantifying HbA2 using techniques such as capillary electrophoresis or high-performance liquid chromatography is effective in detecting most β-thalassemia traits, identifying α-thalassemia traits remains challenging owing to limited screening tests (Han et al., 2019; Mustafa et al., 2020; Jiang et al., 2021; Vachhani et al., 2022). In certain cases, such as individuals with borderline hypochromia, compound triplicated α-globin genes, or β-thalassemia, α^0^-thalassemia may remain undetected (Sorour et al., 2007; Colaco and Nadkarni, 2021).

Two In China, thalassemia screening strategies have been implemented in high-prevalence regions, including Guangxi, Guangdong, Hainan, Yunnan, Fujian, Hong Kong, and Taiwan (Jiang et al., 2021). Among these regions, Guangxi Province has the highest prevalence, and Hb Barts hydrops fetalis constitutes a significant birth defect within the region. Over the course of a decade, severe cases of α-thalassemia have been successfully eliminated in Guangxi Province since the comprehensive implementation of thalassemia prevention and control strategies in 2010. This study aimed to evaluate the effectiveness of current molecular testing strategies for thalassemia and provide insights into screening and diagnosing high-risk couples expecting a child with Hb Barts hydrops fetalis.

## 2 Methods

### 2.1 Subjects and hematological screening strategy

**FIGURE 1:**
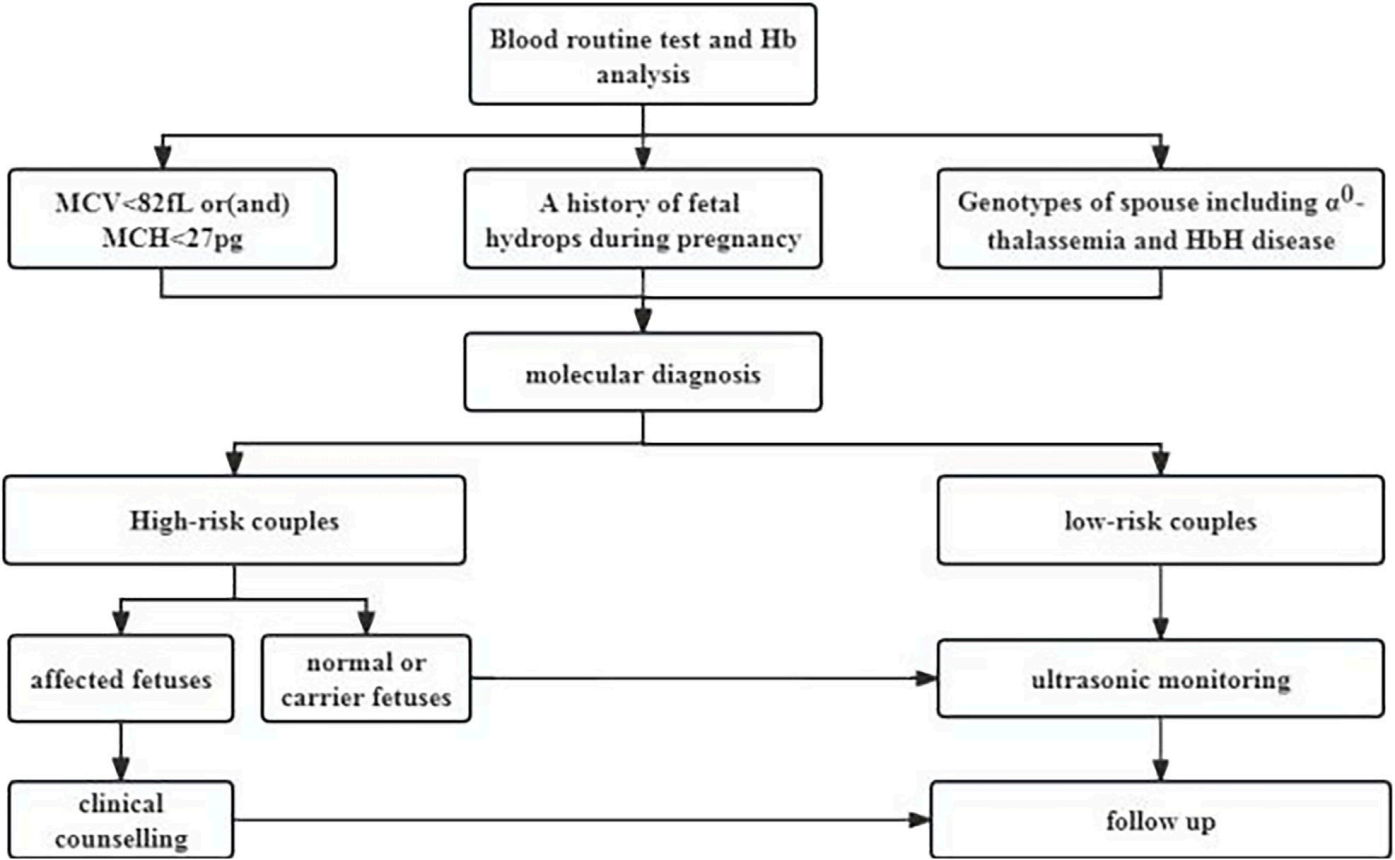
Intervention Strategies, for α-Thalassemia in Individuals of Childbearing Age (Mean corpuscular volume, MCV, Mean corpuscular hemoglobin, MCH; Hemoglobin,Hb).

The prenatal thalassemia intervention program was implemented through a three-level network of maternal and child health services in Guangxi with government support (Figure 1). During the period from 2010 to 2019, an extensive cohort of 91,852 cases derived from 20 districts was meticulously scrutinized for this comprehensive study. Notably, among this cohort, a significant proportion of 49,880 cases pertained to the female population, with a median age of 28 years (ranging from 16 to 49 years). The distribution of pregnancy stages within this cohort revealed that 44.20% of the cases were in the early gestational phase, while the remaining 55.80% were in the mid-pregnancy stage. Additionally, the prevalence rates of severe, moderate, and mild anemia were observed to be 0.06%, 6.74%, and 58.47% respectively. All patients underwent routine Hb variant and thalassemia screening, which included hematology and Hb analysis. Molecular testing for α-thalassemia was recommended when the screening results indicated a mean corpuscular volume (MCV) < 82 fL, mean corpuscular hemoglobin (MCH) < 27 pg, totaling 78,302 cases. Even if the screening results were normal, molecular testing was still recommended for genetic counseling if there was a history of fetal hydrops during pregnancy (7,824 cases) or if either spouse was a carrier of α^0^-thalassemia or Hb H disease (4,726 cases), totaling 13,550 cases. All the patients provided written informed consent.

### 2.2 Genotype test

Genomic DNAs was extracted from the peripheral blood leukocytes of subjects using a DNA blood extraction kit. Gap-PCR was used to identify common Chinese α-globin gene deletion mutations (-α^3.7^, -α^4.2^, and--^SEA^). Reverse dot-blot hybridization was employed to detect three types of α-thalassemia, α^WS^α, α^CS^α, and α^QS^α, as well as 17 types of β-globin gene mutations. In cases of new mutations, gross deletions in the α-gene cluster were detected using multiplex ligation-dependent probe amplification (MLPA). Additionally, DNA sequencing was performed to identify the unknown and rare α-globin mutations. Couples in which both partners carry α^0^-gene mutations are deemed to be at a high risk for severe α-thalassemia in the fetus, whereas couples without such mutations are classified as having a low risk.

### 2.3 Prenatal diagnosis

In couples with α^0^-thalassemia, pregnancy is considered high risk. Prior to prenatal diagnosis, couples designated as at-risk for pregnancy undergo detailed interviews, particularly regarding the risks of invasive sampling, surgical complications, fetal loss rates, and the option to terminate pregnancy if the fetus is diagnosed with Hb Bart’s hydrops fetalis. Written informed consent was obtained from all pregnant women. Invasive examination techniques include chorionic villus sampling (CVS) in early pregnancy (11–14 weeks) and amniocentesis in mid-pregnancy (18–28 weeks), both of which are performed under ultrasonographic guidance. All pregnant women receiving antenatal care at this hospital underwent consecutive ultrasound examinations at 12–15 weeks, 16–20 weeks, and 25–30 weeks to evaluate the presence of fetal edema (Li et al., 2015). For low-risk pregnant women with fetal edema, prenatal diagnosis is recommended to determine the cause of fetal edema through chromosomal analysis, genetic microarray testing, and thalassemia gene testing.

### 2.4 Analysis

Statistical analyses were performed using SPSS version 26. Chi-squared test was used to compare the different types of thalassemia, with the significance level set at *p* < 0.05. Receiver Operating Characteristic (ROC) curve analysis was conducted to determine the optimal cutoff values for MCV and MCH, and the sensitivity (Sen), specificity (Spe), positive predictive values (PPV), and negative predictive values (NPV) of each cutoff were calculated.

## 3 Results

### 3.1 Gene mutation spectrum of α-thalassemia

Among the 91,852 subjects, 38,379 (41.78%) had α-thalassemia mutations, including 34,420 (37.47%) with α-thalassemia and 3,959 (4.31%) with α/β-thalassemia. Out of the 34,420 subjects with α-thalassemia, 20,591 (59.82%) had α^0^-thalassemia and Hb H. Forty-nine genotypes were identified, with the most frequent accounting for 94.27% of all the genotypes, including--^SEA^/αα, -α^3.7^/αα, α^CS^α/αα, -α^4.2^/αα, α^WS^α/αα, --^SEA^/-α^3.7^, and α^QS^α/αα. Three rare α^0^-thalassemia and five rare Hb H disease genotypes were identified: --^THAI^/αα, --^32.8^/αα, --^230^/αα, --^THAI^/-α^3.7^, --^THAI^/α^WS^α, --^THAI^/-α^4.2^, --^THAI^/α^CS^α, and--^SEA^/HKαα (Table 1).

**TABLE 1:** Distribution of genotype among the 34,420 persons with α-thalassemia.

Genotype	Number	Phenotype	Frequency (%)	Genotype	Number	Phenotype	Frequency (%)
--^SEA^/αα	18,881	α^0^/α	54.85	--^THAI^/-α^3.7^	8	α^0^/α^+^	0.023
-α^3.7^/αα	5,336	α^+^/α	15.5	HKαα/αα	8	α^+^/α	0.023
α^CS^α/αα	2,897	α^+^/α	8.41	α^CS^α/α^QS^α	5	α^+^/α^+^	0.014
-α^4.2^/αα	2,400	α^+^/α	6.97	--^THAI^/α^WS^α	3	α^0^/α^+^	0.008
α^WS^α/αα	1769	α^+^/α	5.14	--^THAI^/α^CS^α	3	α^0^/α^+^	0.008
--^SEA^/-α^3.7^	674	α^0^/α^+^	1.96	α^CD37^α/αα	2	α^+^/α	0.006
α^QS^α/αα	459	α^+^/α	1.33	α^CD30^α/αα	2	α^+^/α	0.006
--^SEA^/-α^4.2^	319	α^0^/α^+^	0.93	--^THAI^/-α^4.2^	2	α^0^/α^+^	0.006
--^SEA^/α^WS^α	308	α^0^/α^+^	0.90	--^SEA^/HKαα	2	α^0^/α^+^	0.006
--^SEA^/α^CS^α	236	α^0^/α^+^	0.69	--^230^/αα	2	α^0^/α	0.006
-α^3.7^/-α^3.7^	172	α^+^/α^+^	0.50	α^fusion^α/αα	2	α^+^/α	0.006
-α^3.7^/-α^4.2^	161	α^+^/α^+^	0.47	--^32.8^/αα	1	α^0^/α	0.003
-α^3.7^/α^WS^α	149	α^+^/α^+^	0.43	α^J−Wenchang-Wuming^α/αα	1	α^+^/α	0.003
--^THAI^/αα	140	α^0^/α	0.41	α^Hekinan II^α/αα	1	α^+^/α	0.003
-α^3.7^/α^CS^α	118	α^+^/α^+^	0.34	α^Hb Rus^α/αα	1	α^+^/α	0.003
α^CS^α/α^WS^α	68	α^+^/α^+^	0.2	α^CS^α/HKαα	1	α^+^/α	0.003
-α^4.2^/α^WS^α	65	α^+^/α^+^	0.19	α^CAP+32^α/αα	1	α^+^/α	0.003
-α^4.2^/α^CS^α	58	α^+^/α^+^	0.17	α^C.99^α/αα	1	α^+^/α	0.003
-α^4.2^/-α^4.2^	41	α^+^/α^+^	0.12	-α^8^/αα	1	α^+^/α	0.003
α^CS^α/α^CS^α	31	α^+^/α^+^	0.09	α^3’UTR+51-+53^α/αα	1	α^+^/α	0.003
α^QS^α/α^WS^α	28	α^+^/α^+^	0.08	-α^3.7^/α^fusion^α	1	α^+^/α^+^	0.003
-α^3.7^/α^QS^α	23	α^+^/α^+^	0.067	-α^21.9^/αα	1	α^+^/α	0.003
α^WS^α/α^WS^α	14	α^+^/α^+^	0.041	-α^2.4^/αα	1	α^+^/α	0.003
--^SEA^/α^QS^α	12	α^0^/α^+^	0.035	-α^2.4^/α^Binyang^α	1	α^+^/α^+^	0.003
-α^4.2^/α^QS^α	9	α^+^/α^+^	0.026	Total	34,420		100.00

### 3.2 Gene mutation spectrum of α/β-thalassemia

Among the 3,959 cases of α/β-thalassemia, 1,650 (41.68%) were diagnosed with β-thalassemia combined with α^0^-thalassemia and Hb H disease. A total of 137 genotypes were identified, with the most prevalent accounting for 58.70% of all the genotypes, including β^CD41-42^/β^N^ combined with --^SEA^/αα, -α^3.7^/αα, α^WS^α/αα, -α^4.2^/αα, and α^CS^α/αα. Other common genotypes included β^CD17^/β^N^ combined with --^SEA^/αα and -α^3.7^/αα (Table 2). Additionally, four rare α/β-thalassemia genotypes were identified: ---^THAI^/-α^3.7^, β^CD17^/β^N^; --^THAI^/αα, β^CD17^/β^N^; --^THAI^/αα, β^CD41-42^/β^N^; and --^THAI^/αα, β^−28^/β^N^. Moreover, two α^0^ with β^0^/β^+^ genotypes were identified, namely, --^SEA^/αα, β^CD41-42^/β^CD26^ (0.07%) and --^SEA^/αα, β^CD41-42^/β^−28^ (0.05%), and one genotype, --^SEA^/-α^3.7^, β^−28^/β^IVS−I−1^ (0.03%).

**TABLE 2:** Distribution of genotype among the 3,959 persons with α/β-thalassemia.

Genotype	Number	Phenotype	Frequency (%)	Genotype	Number	Phenotype	Frequency (%)
--^SEA^/αα,β^CD41-42^/β^N^	653	α^0^/α/β^0^/β	16.49	--^THAI^/-α^3.7^,β^CD17^/β^N^	3	α^0^/α^+^/β^0^/β^N^	0.07
--^SEA^/αα,β^CD17^/β^N^	428	α^0^/α/β^0^/β	10.81	--^SEA^/αα,β^CD41-42^/β^CD26^	3	α^0^/α/β^0^/β^+^	0.07
-α^3.7^/αα,β^CD41-42^/β^N^	417	α^+^/α/β^0^/β	10.53	--^SEA^/αα,β^CD14-15^/β^N^	3	α^0^/α/β^0^/β	0.07
-α^3.7^/αα,β^CD17^/β^N^	261	α^+^/α/β^0^/β	6.59	--^SEA^/-α^4.2^,β^−28^/β^N^	3	α^0^/α^+^/β^+^/β	0.07
α^WS^α/αα,β^CD41-42^/β^N^	215	α^+^/α/β^0^/β	5.43	α^CS^α/α^WS^α,β^−28^/β^N^	2	α^+^/α^+^/β^+^/β	0.05
-α^4.2^/αα,β^CD41-42^/β^N^	170	α^+^/α/β^0^/β	4.29	-α^4.2^/αα,β^−29^/β^N^	2	α^+^/α/β^+^/β	0.05
α^CS^α/αα,β^CD41-42^/β^N^	166	α^+^/α/β^0^/β	4.19	-α^4.2^/α^WS^α,β^CD17^/β^N^	2	α^+^/α^+^/β^0^/β	0.05
α^WS^α/αα,β^CD17/^β^N^	132	α^+^/α/β^0^/β	3.33	-α^4.2^/α^CS^α,β^CD17^/β^N^	2	α^+^/α^+^/β^0^/β	0.05
-α^4.2^/αα,β^CD17^/β^N^	129	α^+^/α/β^0^/β	3.25	-α^3.7^/α^WS^α,β^IVS−II−654^/β^N^	2	α^+^/α^+^/β^0^/β	0.05
α^CS^α/αα,β^CD17^/β^N^	115	α^+^/α/β^0^/β	2.90	-α^3.7^/α^WS^α,β^CD17^/β^N^	2	α^+^/α^+^/β^0^/β	0.05
--^SEA^/αα,β^−28^/β^N^	108	α^0^/α/β^+^/β	2.72	-α^3.7^/α^CS^α,β^CD26^/β^N^	2	α^+^/α^+^/β^+^/β	0.05
--^SEA^/αα,β^IVS−II−654^/β^N^	99	α^0^/α/β^0^/β	2.50	-α^3.7^/α^CS^α,β^CD41-42^/β^N^	2	α^+^/α^+^/β^0^/β	0.05
--^SEA^/αα,β^CD26^/β^N^	87	α^0^/α/β^+^/β	2.19	-α^3.7^/-α^4.2^,β^IVS−I−1^/β^N^	2	α^+^/α^+^/β^0^/β	0.05
--^SEA^/αα,β^CD71-72^/β^N^	81	α^0^/α/β^0^/β	2.04	-α^3.7^/-α^3.7^,β^CD26^/β^N^	2	α^+^/α^+^/β^+^/β	0.05
-α^3.7^/αα,β^−28^/β^N^	62	α^+^/α/β^+^/β	1.56	--^THAI^/αα,β^CD17^/β^N^	2	α^+^/α^+^/β^+^/β	0.05
-α^3.7^/αα,β^CD26^/β^N^	51	α^+^/α/β^+^/β	1.28	----^SEA^/αα,β^CD41-42^/β^−28^	2	α^0^/α/β^0^/β^+^	0.05
-α^3.7^/αα,β^CD71-72^/β^N^	49	α^+^/α/β^0^/β	1.23	--^SEA^/α^WS^α,β^CD26^/β^N^	2	α^0^/α^+^/β^+^/β	0.05
-α^3.7^/αα,β^IVS−I−1^/β^N^	41	α^+^/α/β^0^/β	1.03	--^SEA^/α^QS^α,β^CD41-42^/β^N^	2	α^0^/α^+^/β^0^/β	0.05
-α^3.7^/αα,β^IVS−II−654^/β^N^	40	α^+^/α/β^0^/β	1.01	--^SEA^/-α^4.2^,β^IVS−I−1^/β^N^	2	α^0^/α^+^/β^0^/β	0.05
--^SEA^/αα,β^IVS−I−1^/β^N^	34	α^0^/α/β^0^/β	0.86	α^WS^α/αα,β^−29^/β^N^	1	α^+^/α/β^+^/β	0.03
-α^4.2^/αα,β^−28^/β^N^	33	α^+^/α/β^+^/β	0.83	α^WS^α/αα,β^CD17^/β^CD26^	1	α^+^/α/β^0^/β^+^	0.03
α^WS^α/αα,β^−28^/β^N^	32	α^+^/α/β^+^/β	0.80	α^QS^α/αα,β^IVS−II−654^/β^N^	1	α^+^/α/β^0^/β	0.03
α^CS^α/αα,β^−28^/β^N^	30	α^+^/α/β^+^/β	0.75	α^QS^α/αα,β^CD26^/β^N^	1	α^+^/α/β^+^/β	0.03
α^WS^α/αα,β^CD26^/β^N^	29	α^+^/α/β^+^/β	0.73	α^QS^α/αα,β^CD41-42^/β^CD26^	1	α^+^/α/β^0^/β^+^	0.03
α^WS^α/αα,β^CD71-72^/β^N^	28	α^+^/α/β^0^/β	0.71	α^QS^α/α^WS^α,β^CD41-42^/β^N^	1	α^+^/α^+^/β^0^/β	0.03
--^SEA^/-α^3.7^,β^CD41-42^/β^N^	22	α^0^/α^+^/β^0^/β	0.56	α^QS^α/α^WS^α,β^CD17^/β^N^	1	α^+^/α^+^/β^0^/β	0.03
α^WS^α/αα,β^IVS−I−1^/β^N^	21	α^+^/α/β^0^/β	0.53	α^WS^α/α^WS^α,β^CD41-42^/β^N^	1	α^+^/α^+^/β^0^/β	0.03
-α^4.2^/αα,β^CD26^/β^N^	18	α^+^/α/β^+^/β	0.45	α^CS^α/αα,β^IVS−II−654^/β^CD26^	1	α^+^/α/β^0^/β^+^	0.03
α^QS^α/αα,β^CD41-42^/β^N^	17	α^+^/α/β^0^/β	0.43	α^CS^α/αα,β^CD41-42^/β^CD26^	1	α^+^/α/β^0^/β^+^	0.03
α^CS^α/αα,β^IVS−I−1^/β^N^	17	α^+^/α/β^0^/β	0.43	α^CS^α/αα,β^−29^/β^N^	1	α^+^/α/β^+^/β	0.03
-α^4.2^/αα,β^CD71-72^/β^N^	16	α^+^/α/β^0^/β	0.40	α^CS^α/α^WS^α,β^IVS−I−1^/β^N^	1	α^+^/α^+^/β^0^/β	0.03
--^SEA^/α^CS^α,β^CD41-42^/β^N^	15	α^0^/α^+^/β^0^/β	0.38	α^CS^α/α^WS^α,β^CD43^/β^N^	1	α^+^/α^+^/β^0^/β	0.03
-α^4.2^/αα,β^IVS−II−654^/β^N^	14	α^+^/α/β^0^/β	0.35	α^CS^α/α^WS^α,β^CD41-42^/β^N^	1	α^+^/α^+^/β^0^/β	0.03
--^SEA^/αα,β^CD43^/β^N^	14	α^0^/α/β^0^/β	0.35	α^CS^α/α^QS^α,β^−28^/β^N^	1	α^+^/α^+^/β^+^/β	0.03
α^WS^α/αα,β^IVS−II−654^/β^N^	13	α^+^/α/β^0^/β	0.33	α^CS^α/α^CS^α,β^CD41-42^/β^N^	1	α^+^/α^+^/β^0^/β	0.03
α^CS^α/αα,β^IVS−II−654^/β^N^	13	α^+^/α/β^0^/β	0.33	α^CS^α/α^CS^α,β^−28^/β^N^	1	α^+^/α^+^/β^+^/β	0.03
α^CS^α/αα,β^CD26^/β^N^	12	α^+^/α/β^+^/β	0.30	α^CS^α/-α^3.7^,β^CD17^/β^N^	1	α^+^/α^+^/β^0^/β	0.03
--^SEA^/-α^3.7^,β^CD17^/β^N^	12	α^0^/α^+^/β^0^/β	0.30	-α^4.2^/αα,β^−90^/β^N^	1	α^+^/α/β^+^/β	0.03
--^SEA^/α^CS^α,β^CD17^/β^N^	11	α^0^/α^+^/β^0^/β	0.28	-α^4.2^/-α^4.2^-,β^CD26^/β^N^	1	α^+^/α^+^/β^+^/β	0.03
α^QS^α/αα,β^CD17^/β^N^	10	α^+^/α/β^0^/β	0.25	-α^4.2^/-α^4.2^,β^CD71-72^/β^N^	1	α^+^/α^+^/β^0^/β	0.03
-α^4.2^/αα,β^IVS−I−1^/β^N^	10	α^+^/α/β^0^/β	0.25	-α^4.2^/-α^4.2^-,β^CD41-42^/β^N^	1	α^+^/α^+^/β^0^/β	0.03
-α^3.7^/αα,β^CD43^/β^N^	10	α^+^/α/β^0^/β	0.25	-α^4.2^/-α^4.2^,β^CD17^/β^N^	1	α^+^/α^+^/β^0^/β	0.03
--^SEA^/-α^4.2^,β^CD17^/β^N^	10	α^0^/α^+^/β^0^/β	0.25	-α^3.7^/αα,β^CD26^/β^CD26^	1	α^+^/α/β^+^/β^+^	0.03
-α^3.7^/α^WS^α,β^CD41-42^/β^N^	9	α^+^/α^+^/β^0^/β	0.23	-α^3.7^/αα,β^CD41-42^/β^CD17^	1	α^+^/α/β^0^/β^0^	0.03
--^SEA^/-α^4.2^,β^CD41-42^/β^N^	9	α^0^/α^+^/β^0^/β	0.23	-α^3.7^/αα,β^−28^/β^CD27/28^	1	α^+^/α/β^+^/β^0^	0.03
α^CS^α/αα,β^CD71-72^/β^N^	8	α^+^/α/β^0^/β	0.2	-α^3.7^/α^WS^α,β^CD71-72^/β^N^	1	α^+^/α^+^/β^0^/β	0.03
--^SEA^/α^WS^α,β^CD41-42^/β^N^	8	α^0^/α^+^/β^0^/β	0.2	-α^3.7^/α^WS^α,β^CD43^/β^N^	1	α^+^/α^+^/β^0^/β	0.03
--^SEA^/α^WS^α,β^CD17^/β^N^	7	α^0^/α^+^/β^0^/β	0.17	-α^3.7^/α^QS^α,β^CD26^/β^N^	1	α^+^/α^+^/β^+^/β	0.03
-α^3.7^/αα,β^−29^/β^N^	6	α^+^/α/β^+^/β	0.15	-α^3.7^/α^QS^α,β^CD71-72^/β^N^	1	α^+^/α^+^/β^0^/β	0.03
-α^3.7^/-α^4.2^,β^CD41-42^/β^N^	6	α^+^/α^+^/β^0^/β	0.15	-α^3.7^/α^QS^α,β^CD41-42^/β^N^	1	α^+^/α^+^/β^0^/β	0.03
--^SEA^/αα,β^−29^/β^N^	6	α^0^/α/β^+^/β	0.15	-α^3.7^/α^CS^α,β^IVS−I−1^/β^N^	1	α^+^/α^+^/β^0^/β	0.03
β^CD43^/β^N^	5	α^+^/α/β^0^/β	0.12	-α^3.7^/α^CS^α,β^CD26^/β^CD26^	1	α^+^/α^+^/β^+^/β^+^	0.03
α^QS^α/αα,β^CD71-72^/β^N^	4	α^+^/α/β^0^/β	0.10	-α^3.7^/-α^3.7^,β^IVS−I−1^/β^N^	1	α^+^/α^+^/β^0^/β	0.03
α^CS^α/α^WS^α,β^CD17/^β^N^	4	α^+^/α^+^/β^0^/β	0.10	-α^3.7^/-α^3.7^,β^CD71-72^/β^N^	1	α^+^/α^+^/β^0^/β	0.03
-β^CD43^/β^N^	4	α^+^/α/β^0^/β	0.10	-α^3.7^/-α^3.7^,β^−28^/β^N^	1	α^+^/α^+^/β^+^/β	0.03
-α^4.2^/αα,β^CD27/28^/β^N^	4	α^+^/α/β^0^/β	0.10	--^THAI^/αα,β^CD41-42^/β^N^	1	α^0^/α/β^0^/β	0.03
-α^4.2^/α^CS^α,β^CD41-42^/β^N^	4	α^+^/α^+^/β^0^/β	0.10	--^THAI^/αα,β^−28^/β^N^	1	α^0^/α/β^+^/β	0.03
-α^3.7^/-α^4.2^,β^CD17^/β^N^	4	α^+^/α^+^/β^0^/β	0.10	--^SEA^/αα,β^CAP+^/β^N^	1	α^0^/α/β^+^/β	0.03
--^SEA^/αα,β^CD27/28^/β^N^	4	α^0^/α^+^/β^0^/β	0.10	--^SEA^/α^WS^α,β^IVS−II−654^/β^N^	1	α^0^/α^+^/β^0^/β	0.03
--^SEA^/α^WS^α,β^CD71-72^/β^N^	4	α^0^/α^+^/β^0^/β	0.10	--^SEA^/α^WS^α,β^−28^/β^N^	1	α^0^/α^+^/β^+^/β	0.03
--^SEA^/-α^3.7^,β^−28^/β^N^	4	α^0^/α^+^/β^+^/β	0.10	--^SEA^/α^CS^α,β^IVS−II−654^/β^N^	1	α^0^/α^+^/β^0^/β	0.03
α^WS^α/α^WS^α,β^CD41-42^/β^N^	3	α^+^/α^+^/β^0^/β	0.07	--^SEA^/α^CS^α,β^CD26^/β^N^	1	α^0^/α^+^/β^+^/β	0.03
α^QS^α/αα,β^−28^/β^N^	3	α^+^/α/β^+^/β	0.07	--^SEA^/α^CS^α,β^−28^/β^N^	1	α^0^/α^+^/β^+^/β	0.03
β^CD43^/β^N^	3	α^+^/α/β^0^/β	0.07	--^SEA^/-α^4.2^,β^CD26^/β^N^	1	α^0^/α^+^/β^+^/β	0.03
-α^4.2^/α^WS^α,β^CD41-42^/β^N^	3	α^0^/α/β^0^/β	0.07	--^SEA^/-α^3.7^,β^CD26^/β^N^	1	α^0^/α^+^/β^+^/β	0.03
-α^3.7^/αα,β^CD27/28^/β^N^	3	α^+^/α^+^/β^0^/β	0.07	--^SEA^/-α^3.7^,β^IVS−I−1^/β^N^	1	α^0^/α^+^/β^0^/β	0.03
-α^3.7^/α^CS^α,β^CD71-72^/β^N^	3	α^+^/α^+^/β^0^/β	0.07	--^SEA^/-α^3.7^,β^−28^/β^IVS−I−1^	1	α^0^/α^+^/β^+^/β^0^	0.03
-α^3.7^/-α^3.7^,β^CD41-42^/β^N^	3	α^+^/α^+^/β^0^/β	0.07	Total	3,959		100
-α^3.7^/-α^3.7^,β^CD17^/β^N^	3	α^+^/α^+^/β^0^/β	0.07				

### 3.3 The status of α-thalassemia

Table 3 illustrates the frequency distribution of mutation types among all α-thalassemia mutant chromosomes based on the identification of twenty-five distinct mutations. Hematology parameters of 10 main types α-thalassemiaand α/β-thalassemia were shown in supplement Table 1. The most predominant mutation observed was--^SEA^, accounting for 53.71% of all α-mutant chromosomes. The most frequent mutations, in descending order, were -α^3.7^, α^CS^α, -α^4.2^, α^WS^α, α^QS^α, and --^THAI^. Additionally, two rare α^0^ mutations, namely, --^32.8^ and --^230^, were observed. Overall, 22,238 (57.94%) patients had α^0^-thalassemia mutations.

**TABLE 3:** Allele frequency of α-thalassemia among 38,379 persons.

Mutation	Phenotype	Number	Frequency (%)
--SEA	α^0^	22,074	53.71
-α3.7	α^+^	7,862	19.13
αCSα	α^+^	3,874	9.43
-α4.2	α^+^	3,553	8.65
αWSα	α^+^	2,958	7.20
αQSα	α^+^	581	1.41
--THAI	α^0^	161	0.39
HKαα	α^+^	11	0.03
αfusionα	α^+^	2	0.006
--230	α^0^	2	0.006
-α2.4	α^+^	2	0.006
αCD37α	α^+^	2	0.006
αCD30α	α^+^	2	0.006
α^C.99^α	α^+^	1	0.002
-α8	α^+^	1	0.002
--32.8	α^0^	1	0.002
αBinyangα	α^+^	1	0.002
-α21.9	α^+^	1	0.002
α3′UTR+51-+53α	α^+^	1	0.002
αCAP+32α	α^+^	1	0.002
αHb Rusα	α^+^	1	0.002
αJ-Wenchang-Wumingα	α^+^	1	0.002
αHekinan IIα	α^+^	1	0.002
Total		41,096	100.00

A total of 2,235 high-risk couples were identified, 562 of which were affected. Among these patients, three had the--^SEA^/--^THAI^ genotype and one had the--^SEA^/--^230^ genotype. All the affected patients opted for pregnancy termination. Additionally, we observed four patients with fetal anemia and/or mild edema, as well as two cases of severe fetal edema during prenatal diagnosis. The chromosomal and genetic microarray results were normal. Thalassemia gene testing revealed an α^CS^α/α^CS^α genotype in four patients with anemia and/or mild edema, whereas two patients with severe fetal edema had one--^SEA^/α^CS^α genotype and one--^SEA^/--^GX^ genotype. The status of screening and diagnosis for thalassemia from 2010 to 2019 were shown in supplement Table 2.

### 3.4 Evaluation of α-thalassemia genetic test

**FIGURE 2:**
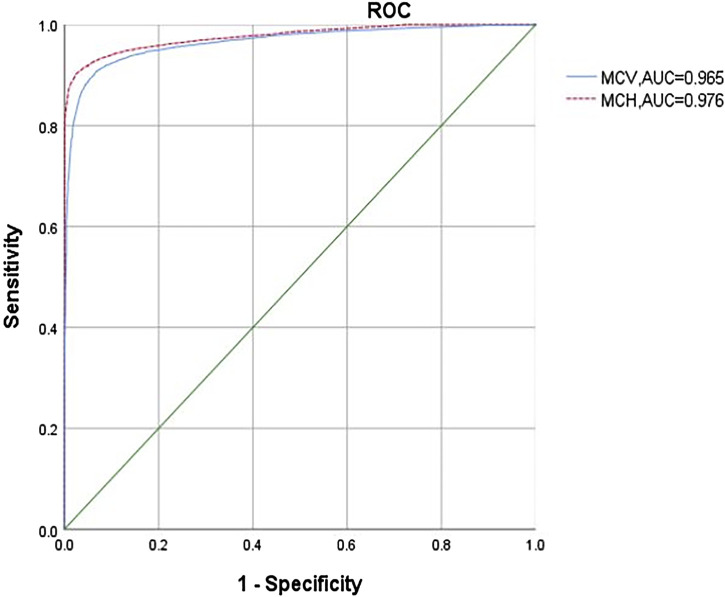
ROC analysis of MCV (Mean corpuscular volume) and MCH (Mean corpuscular hemoglobin) in diagnosing α^0^-thalassemia.

In our study, 13,550 patients had an MCV of >82 fL and an MCH of >27 pg. Among them, 8% (1,084 cases) were carriers of α-thalassemia, including -α^3.7^/αα (40.96%), α^WS^α/αα (34.50%), -α^4.2^/αα (18.73%), α^CS^α/αα (3.78%), --^SEA^/αα (0.37%) (one patient had β^CD26^/β^N^), α^QS^α/αα (0.18%), and others (1.48%). ROC curves demonstrated that MCV and MCH could effectively differentiate between α^0^-thalassemia and α^+^-thalassemia carriers, with optimal cut-off points of 74.6 fL (The Sen, Spe, PPV, and NPV was 0.963, 0.925, 0.804, and 0.987, respectively.) and 24.4 pg (The Sen, Spe, PPV, and NPV was 0.977, 0.905, 0.767, and 0.992, respectively.), respectively (AUC of 0.965 and 0.976, respectively; Figure 2). Using these cutoff points as criteria for identifying α^0^-thalassemia carriers and HbH disease, only 69,146 cases required genetic testing for α-thalassemia. This would result in a cost-saving of 10,217,700 ¥ based on the cost of the kit (with an average cost of 450 ¥ for α-thalassemia genetic testing). Three high-risk couples were excluded from this study. A comparison of the adoption of national standards reveals (Cut-off values of 82 fL (the Sen, Spe, PPV, and NPV was 0.999, 0.912, 0.785, and 0.999, respectively.) and 27 pg (The Sen, Spe, PPV, and NPV was 0.999, 0.937, 0.835, and 0.999, respectively.) for MCV and MCH) no differences in the results.

## 4 Discussion

In Guangxi, the carrier rate of thalassemia genes is approximately 20%, which is significantly higher than that in other provinces and regions, such as Guangdong (11.6%), Hong Kong (8.4%), Taiwan (5.5%), Sichuan (4.1%), and Jiangxi (2.7%) (Liu et al., 2019; Jiang et al., 2021). As a result, fetal hydrops syndrome (severe α-thalassemia) has consistently been a prominent birth defect in the Guangxi region for many years. The Guangxi government has prioritized the prevention and control of thalassemia by implementing a three-level healthcare network strategy that includes policy interventions by government departments (He et al., 2014). Our center, being one of the three provincial-level prenatal diagnosis centers in Guangxi, plays a crucial role in prenatal diagnosis, birth defect management, and quality control in the region. Our center conducts prenatal diagnosis for thalassemia for over 10,000 people annually, accounting for about one-third of thalassemia prenatal diagnoses in the Guangxi region. With the help of the “Guifuer system,” we perform quality control and follow-up on the screening and diagnostic results entered into the system. No newborn with severe α-thalassemia was identified in the follow-up studies.

In China, thalassemia screening recommends using cut-off values of 82 fL and 27 pg for MCV and MCH, respectively (Jiang et al., 2021). Applying these national criteria for screening for α^0^-thalassemia can help identify all patients with HbH disease and the majority with α^0^-thalassemia. In our study, which included both patients with α-thalassemia and α/β-thalassemia, we identified 38,379 patients with α-thalassemia mutations. In this cohort, 57.94% of the patients were classified as having α^0^-thalassemia and Hb H. Of the 2,235 high-risk couples identified, 562 pregnancies were terminated. These findings highlight the effectiveness of molecular screening procedures in identifying high-risk couples.

However, with increasing population migration and the emergence of new gene variants, relying solely on common α-thalassemia screening may fail to detect certain α^0^-gene types, resulting in the birth of children with severe α-thalassemia (Henderson et al., 2016; Lai et al., 2017; Nasiri et al., 2020). To accurately identify high-risk couples, we conducted rare gene testing with informed consent from those who tested negative in routine gene testing. In our study, we identified 18 rare gene types, including two α^0^-thalassemia genes (--^32.8^ and --^230^), excluding the--^THAI^ type. Previous research has indicated a relatively common carrier rate of--^THAI^ (0.36%) in Guangxi (He et al., 2017b). Since 2014, molecular testing for this gene has been routinely conducted. In our study, among 562 affected cases, three had the--^SEA^/--^THAI^ genotype and one had the--^SEA^/--^230^. In practical settings, not every individual who tests negative for routine gene testing agrees to undergo rare gene testing because of the high cost. Additionally, we observed two patients with a prenatal diagnosis attributed to severe fetal edema, and the results of α-thalassemia gene testing revealed one--^SEA^/--^GX^ genotype and one--^SEA^/α^CS^α genotype. This indicates a potential association with severe fetal edema.

The screening program aims to identify individuals with α^0^-thalassemia; however, some patients with α^+^-thalassemia, particularly those with Hb CS, may be missed. Previous studies have reported that Hb H disease, especially the --/α^CS^α and α^CS^α/α^CS^α genotypes, can result in fetal anemia or edema (He et al., 2016; Sirilert et al., 2019). However, our screening program incorporates Hb analysis, specifically utilizing CE technology, which significantly enhances the detection rate of α^CS^α/αα and α^CS^α (Panyasai et al., 2023). In this study, we observed four patients with a prenatal diagnosis attributed to fetal edema or mild anemia, excluding abnormalities in chromosomal and genetic microarray testing results. The α-thalassemia gene testing results showed that four patients had α^CS^α/α^CS^α, indicating a potential association with thalassemia. Pregnant women with Hb H often experience severe anemia during pregnancy (Lal and Vichinsky, 2023). Fetails with genotypes α^QS^α and α^CS^α typically present with moderate anemia, whereas a few patients may have severe anemia that requires blood transfusion therapy. Therefore, as part of antenatal care, we recommend conducting α^+^-thalassemia testing to identify Hb H.

Hematological screening for α-thalassemia can yield false positive and false negative results, leading to fewer missed diagnoses in high-risk couples. In our study, we conducted α-thalassemia gene testing for individuals with a history of fetal edema or cases in which one partner was diagnosed with α^0^-thalassemia or Hb H disease, even if their MCV was >82 fL and MCH was >27 pg. Among the 13,550 patients, 8% were carriers of α-thalassemia, including 4 cases of--^SEA^/αα (0.37%). These results are consistent with those reported by Jiang et al. (Jiang et al., 2021), who did not find any patients with --^SEA^/αα. ROC curves showed that MCV and MCH (cut-off points of 74.6 fL and 24.4 pg) could effectively identify α^0^-thalassemia carriers and HbH disease. By testing only 69,146 cases of α-thalassemia, cost cost-saving of 10,217,700 ¥ are achieved. The number of missed diagnoses in high-risk couples was consistent with the national screening standards.

The study has a number of limitations. Firstly, the data were collected from a single tertiary hospital, representing only a subset of cases in our region. Therefore, the generalizability of the findings to the broader population may be limited due to the specific sample source. Secondly, although rare α-thalassemia gene testing was implemented, there is a potential risk of underreporting certain genetic variants, which could result in the oversight of severe α-thalassemia cases. Thirdly, a comprehensive cost-benefit analysis was not conducted. However, the proposed cutoff points in this study indicate that the implementation of screening using a new parameter has the potential to reduce current expenses. Further research is needed to validate and optimize this strategy and assess its feasibility.

In summary, our study highlights the success of implementing molecular screening procedures to identify cases of severe α-thalassemia and emphasizes the importance of considering α^+^-thalassemia testing for HbH disease detection. This information will be valuable in guiding clinical practice and improving the detection and management of α-thalassemia.

## Data Availability

The original contributions presented in the study are included in the article/Supplementary Material, further inquiries can be directed to the corresponding authors.
