# Supplementary material for: Routine antenatal molecular testing for α-thalassemia at a tertiary referral hospital in China: ten years of experience
Source: Front Genet. 2024 Jun 4;15:1416047. doi: 10.3389/fgene.2024.1416047 (PMC11183328; doi:10.3389/fgene.2024.1416047)
Supplement: Supplementary file 1 [file Table1.docx]

**SUPPLEMENT** TABLE 1 Hematology parameters of 10 main types α-thalassemia and α/β-thalassemia

| Genotype |  | Hb (g/L) | MCV (fL) | MCH (pg) |
| --- | --- | --- | --- | --- |
| α-thalassemia |  |  |  |  |
| --^SEA^/αα | 18881 | 125.29±17.26 | 67.99±4.20 | 21.58±1.67 |
| -α^3.7^/αα | 5336 | 131.59±17.97 | 82.15±4.15 | 26.17±1.63 |
| α^CS^α/αα | 2897 | 128.01±18.35 | 79.70±4.41 | 25.94±1.72 |
| -α^4.2^/αα | 2400 | 132.35±18.38 | 82.23±4.62 | 26.36±1.75 |
| α^WS^α/αα | 1769 | 134.27±19.53 | 82.35±6.77 | 26.70±2.04 |
| --^SEA^/-α^3.7^ | 674 | 96.15±15.28 | 60.58±5.33 | 18.24±1.45 |
| α^QS^α/αα | 459 | 127.65±17.54 | 75.48±6.21 | 24.56±1.54 |
| --^SEA^/-α^4.2^ | 319 | 95.64±14.49 | 61.24±5.54 | 18.26±1.44 |
| --^SEA^/α^WS^α | 308 | 121.10±18.28 | 66.72±3.26 | 21.02±1.18 |
| --^SEA^/α^CS^α | 236 | 88.66±15.94 | 64.66±4.13 | 19.72±1.46 |
| α/β-thalassemia |  |  |  |  |
| --^SEA^/αα,β^CD41-42^/β^N^ | 653 | 123.88±17.71 | 69.59±3.38 | 22.49±1.35 |
| --^SEA^/αα,β^CD17^/β^N^ | 428 | 124.63±17.24 | 68.41±3.45 | 22.01±1.34 |
| -α^3.7^/αα,β^CD41-42^/β^N^ | 417 | 122.10±17.20 | 66.69±3.62 | 21.21±1.26 |
| -α^3.7^/αα,β^CD17^/β^N^ | 261 | 121.66±16.77 | 65.67±3.43 | 20.88±1.16 |
| α^WS^α/αα,β^CD41-42^/β^N^ | 215 | 119.24±16.73 | 63.86±3.95 | 20.13±1.26 |
| -α^4.2^/αα,β^CD41-42^/β^N^ | 170 | 122.33±17.12 | 66.88±3.97 | 21.43±1.49 |
| α^CS^α/αα,β^CD41-42^/β^N^ | 166 | 117.62±17.38 | 67.24±3.82 | 21.37±1.27 |
| α^WS^α/αα,β^CD17/^β^N^ | 132 | 118.66±18.66 | 63.79±3.06 | 20.04±1.93 |
| -α^4.2^/αα,β^CD17^/β^N^ | 129 | 121.79±17.80 | 66.10±3.87 | 21.18±1.29 |
| α^CS^α/αα,β^CD17^/β^N^ | 115 | 119.95±17.07 | 66.68±4.11 | 21.25±1.37 |

**SUPPLEMENT** TABLE 2 The status of screening and diagnosis for thalassemia from 2010 to 2019.

| Year | Number of screen | Number of high-risk of couples | Number of Hb Bart's |
| --- | --- | --- | --- |
| 2010 | 3597 | 62 | 18 |
| 2011 | 8836 | 293 | 72 |
| 2012 | 11826 | 343 | 85 |
| 2013 | 16109 | 494 | 122 |
| 2014 | 10480 | 324 | 84 |
| 2015 | 7106 | 177 | 43 |
| 2016 | 7360 | 156 | 39 |
| 2017 | 7531 | 140 | 32 |
| 2018 | 9394 | 149 | 38 |
| 2019 | 9613 | 115 | 29 |
| Total | 91852 | 2253 | 562 |
